# Yersinia fenwicki sp. nov., isolated from human clinical cases in Aotearoa | New Zealand and Australia

**DOI:** 10.1099/ijsem.0.007034

**Published:** 2026-01-21

**Authors:** Lucia Rivas, Hugo Strydom, Hilary Miller, David Winter, Angela Cornelius, Jing Wang, Rikki Graham, Asha Kakkanat, Gino Micalizzi, Jacqueline (Jackie) Wright

**Affiliations:** 1 Health Security Group, New Zealand Institute for Public Health and Forensic Science, Formerly Institute of Environmental Science and Research (ESR), Christchurch Science Centre, Christchurch, New Zealand; 2 Enteric Reference Laboratory, New Zealand Institute for Public Health and Forensic Science, Upper Hutt, New Zealand; 3 Health Security Group, New Zealand Institute for Public Health and Forensic Science, Kenepuru, New Zealand; 4 Public Health Microbiology, Public and Environmental Health Reference Laboratories, Queensland Department of Health, Coopers Plains, Queensland, Australia

**Keywords:** clinical, *Yersinia fenwicki*, *Yersinia species* nov.

## Abstract

A Gram-negative bacillus isolated from human clinical cases in Aotearoa | New Zealand (NZ) and Australia was identified as a new species within the genus *Yersinia* based on genetic and phenotypic characteristics. This species demonstrated distinct biochemical differences in comparison to those typically reported for *Yersinia enterocolitica* (biotypes 1A, 1B, 2, 3, 4 and 5), including the ability to utilise raffinose and melibiose. Whole-genome sequencing data identified a total of 11 strains as a novel multi locus sequence type 598 (using the seven-gene McNally scheme). A closed genome for this species was obtained using Oxford Nanopore long-read sequencing, polished with high-accuracy Illumina short-read sequence data. Analysis of the 16S rRNA showed the closest similarity (98.36%) to *Yersinia hibernica* and *Yersinia artesiana*. Average nucleotide identity (ANI) values were below the threshold of ≤95% ANI when compared to the type strains of other *Yersinia* species, with *Y. enterocolitica* subsp. *enterocolitica* (94.1%) identified as the closest relative, thereby providing evidence that these strains should be considered as a novel species. The type strain is NZRM 4767^T^*=*DSM 120367^T^.

## Introduction

The genus *Yersinia* is a member of the order *Enterobacterales* and belongs to the family *Yersiniaceae* [1]. Currently, there are 26 species within the genus *Yersinia*. Whilst many species within *Yersinia* are considered to be of low pathogenicity to humans and found within the environment, there are at least three species including *Yersinia enterocolitica*, *Yersinia pestis* (the causative agent of plague) and *Yersinia pseudotuberculosis* which are pathogenic to immunocompetent humans [2]. These three species have been shown to have evolved independently [3].

In Aotearoa | New Zealand (NZ), *Y. enterocolitica* and less frequently *Y. pseudotuberculosis* cause significant gastrointestinal infection in human and animals with no recorded cases of *Y. pestis* [4]. There are six internationally recognised *Y. enterocolitica* biotypes (BTs) (1A, 1B, 2, 3, 4 and 5), which can be further subdivided into numerous (>48) serotypes based on their heat-stable antigens [5,6]. *Y. enterocolitica* BT 1A, which includes a wide range of serotypes, is often considered internationally as non-pathogenic because it lacks the classical *Y. enterocolitica* virulence determinants that include a virulence plasmid (pYV) and chromosomal gene invasin (*inv*), as well as the attachment and invasion locus (*ail*) [7,8]. However, growing epidemiological evidence suggests that *Y. enterocolitica* BT 1A can cause gastrointestinal disease [9].

The NZ case definition for a confirmed human case of yersiniosis, which includes YE BT 1A, is a clinically compatible illness accompanied by laboratory definitive evidence of either (a) isolation of *Y. enterocolitica* or *Y. pseudotuberculosis* from blood or faeces, or (b) detection of *Yersinia* spp. nucleic acid from faeces [10]. All diagnostic laboratories in NZ are required to reflex culture all *Yersinia* screen-positive samples and forward isolates to the national Enteric Reference Laboratory (ERL) for confirmation and epidemiological typing. Following a detailed validation process, which included parallel testing of several hundred isolates, routine whole-genome sequencing of all *Yersinia* isolates referred to ERL was implemented for species identification and further typing in 2024.

In 2010, the ERL first observed an atypical *Yersinia* strain from a human faecal sample referred from a primary clinical diagnostic laboratory. Biochemical typing of the strain showed biochemical reactions that differentiated it from *Y. enterocolitica*. However, the phenotypic characteristics observed were similar to those previously reported as unusual *Y. enterocolitica* strains recovered from domestic animals and clinical cases in NZ in the 1990s [11]. Therefore, this strain was originally reported as ‘atypical *Y. enterocolitica*’. Unfortunately, the strains from the study of Fenwick *et al*. [11] were not available for the current analysis. The genomes of six of the atypical *Y. enterocolitica* isolated from clinical samples were submitted to EnteroBase *Yersinia* and assigned a sequence type (ST) 598 (McNally scheme [12,13]) and hierarchical cluster (HierCC) [14] Yersinia HC1490_4399, which has been reported as a novel species of *Yersinia* [15]. In 2023, an additional two genomes identifying as ST598 and *Yersinia* HC1490_4399 were submitted into the EnteroBase *Yersinia* database by an Australian laboratory following isolation from human clinical samples. These isolates were identified from Australian human clinical samples tested in 2023 and characterised as *Y. enterocolitica* BT 1A using biochemical typing.

This study aimed to describe a new species within *Yersinia* with the support of genomic and biochemical data. As this novel species has a similar biochemical profile as the unusual YE first described by Fenwick *et al*. [11], and in recognition of the contribution Emeritus Professor Stanley Fenwick has made to the study of *Yersinia* in NZ, we propose that this novel species be named *Yersinia fenwicki*.

## Physiology and chemotaxonomy

Table 1 outlines the bacterial isolates and genomic data analysed in this study. One of the NZ clinical isolates has been assigned the type strain for *Y. fenwicki* and has been deposited in the New Zealand Reference Culture Medical Section (NZRM) as NZRM 4767^T^ and in the Deutsche Sammlung von Mikroorganismen und Zellkulturen (DSMZ) as DSM 120367^T^. MALDI-TOF MS [Bruker MALDI Biotyper^®^ (MBT); Billerica, MA, USA] identified NZRM 4767^T^ as *Y. enterocolitica/Yersinia intermedia*. The protein patterns of all the strains tested matched with patterns already known for *Yersinia* species with scores of between 1.77 and 2.16 (MBT Compass Library 2023). Therefore, this strain cannot be distinguished from its genetically closest relatives using this technique [16].

**Table 1.** Isolates belonging to *Y. fenwicki* sp. nov. The New Zealand Reference Medical Section (NZRM) number is shown for isolates deposited into that collection. The numbers in the brackets are the PHF Science Laboratory numbers. The type strain for *Y. fenwicki* sp. nov. is denoted with ^T^ and is equivalent to the DSMZ GmBH collection DSM 120367^T^. The complete genomes of NZRM 4767^T^ and raw sequence reads for all NZ and Australian isolates analysed in this study are available in the National Center for Biotechnology Information (NCBI) Short Read Archive (SRA) under BioProjects PRJNA1142067 and PRJEB83896, respectively

Isolate no.	Isolation information				Biosample accession
	Source	Material	Country	Year	
NZRM 4767^T^ (15ER0244)	Human	Faecal	New Zealand	2015	SAMN43575973
NZRM 4765 (15ER0058)	Human	Faecal	New Zealand	2015	SAMN43575971
NZRM 4766 (15ER0187)	Human	Faecal	New Zealand	2015	SAMN43575972
NZRM 4768 (18ER0470)	Human	Faecal	New Zealand	2018	SAMN43575974
NZRM 4769 (18ER0612)	Human	Faecal	New Zealand	2018	SAMN43575975
NZRM 4770 (21ER3874)	Human	Faecal	New Zealand	2021	SAMN44268530
NZRM 4771 (22ER0879)	Human	Faecal	New Zealand	2022	SAMN43575976
22ER4185	Human	Faecal	New Zealand	2022	SAMN43575977
NZRM4780 (24ER0337)	Human	Faecal	New Zealand	2024	SAMN43575978
M2300673ta	Human	Faecal	Australia	2023	SAMEA117565739
M2301254ta	Human	Faecal	Australia	2023	SAMEA117565740

Biochemical profiling as previously described [16] was performed for NZRM 4767^T^ and all associated ST598 isolates that were viable in NZ (Table 1). Bacterial cells were aerobically cultured on Tryptone Soya Agar (TSA; Fort Richard, Auckland, NZ) and Cefsulodin-Irgasan-Novobiocin (CIN; Fort Richard) agar plates and incubated at 28 °C for 18 h±2 h. Gram staining was performed using colonies from TSA, and cell morphology was observed using an optical microscope. Both API 20E and API 50 CH kits (bioMérieux; Marcy-l’Étoile, France) were inoculated and incubated at 28 °C to determine biochemical characteristics and sugar fermentation, respectively. In addition, motility was tested using in-house motility test media in a stab tube, and a lipid hydrolysis test was performed using Nagler agar plates (Fort Richard, Auckland, NZ) inoculated and incubated at 28 °C for 48 h. Catalase activity was considered positive when bubble production in 3% (v/v) hydrogen peroxide solution was observed. Oxidase activity was evaluated using filter paper moistened with tetramethyl-*p*-phenylenediamine (Becton, Dickinson and Company, Franklin Lakes, NJ, USA).

NZRM 4767^T^ demonstrated positive reactions for both the aesculin and salicin biochemical tests, which are two reactions that are used to differentiate YE BT 1A from BTs 1B–5 [5] (Table 2). However, distinct biochemical differences were also observed for NZRM 4767^T^ in comparison to those typically reported for all YE (Table 2), including the ability to utilise raffinose and melibiose and an inability to decarboxylate ornithine. All other associated NZ ST598 isolates tested demonstrated the same biochemical test results as NZRM 4767^T^. Using a *Y. enterocolitica* biotyping scheme adapted from Wauters *et al*. [5], both Australian isolates exhibited fermentation of xylose, trehalose and salicin; production of indole; and tween-esterase and pyrazinamidase activity at 25 °C after 48 h which correlated with *Y. enterocolitica* BT 1A. As reported in other studies, identification of *Yersinia* to the species level by traditional biochemical methods can be difficult due to heterogeneous biochemical phenotypes [17,19].

**Table 2.** Biochemical results for NZRM 4767^T^ *Y. fenwicki* compared with those of other *Yersinia species* with >90 % average nucleotide identity (ANI) similarity and/or >98 % pairwise similarities with the 16S rRNA gene for NZRM 4767^T^ *Y. fenwicki*. 1, *Y. fenwicki* sp. nov. (n=8); 2, *Y. enterocolitica* (YE) biotype (BT) 1A (n=27); 3, YE BT 1B (n=4); 4, YE BT 2 (n=58); 5, YE BT 3 (n=23); 6, YE BT 4 (n=30); 7, YE BT 5 (n=4); 8, *Yersinia artesiana* (n=4*)*; 9, *Yersinia proxima* (n=10); 10, *Yersinia hibernica* (n=2). Results for YE, *Y. artesiana*, *Y. proxima* and *Y. hibernica* were extracted from Le Guern *et al*. [16]. Testing for *Y. fenwicki* included eight strains from NZ only (Table 1). Criteria used based on Le Guern *et al*. [16]: +, 90 % or more strains positive; −, 90 % or more strains negative; d, 11–89 % of strains positive. Shaded results are key differentiators for *Y. fenwicki* compared with other *Yersinia* species. All strains were negative for lysine decarboxylase, D-adonitol and L-rhamnose, starch and D-tagatose; and all were positive for urease, sucrose, L-arabinose, D-ribose and D-cellobiose.

Characteristic	1	2	3	4	5	6	7	8	9	10
**Utilisation of:**										
β-Galactosidase	+	+	+	+	+	+	d	+	+	+
Ornithine decarboxylase	**−**	d	d	d	d	−	−	d	d	d
Citrate utilisation	d	−	−	−	−	−	−	−	d	−
Indole	+	+	−	+	−	−	−	+	+	−
Acetoin production	d	+	+	+	d	+	d	+	+	−
API 50 CH panel										
d-Xylose	+	+	+	+	+	−	+	+	+	+
l-Sorbose	+	+	+	+	+	+	d	+	+	+
Inositol	+	+	−	+	d	−	−	+	+	+
d-Sorbitol	+	+	+	+	+	+	d	+	+	+
Arbutin	+	+	+	d	d	d	d	+	+	+
Aesculin ferric citrate	+	+	−	−	−	−	−	+	+	+
Salicin	+	d	−	d	−	−	d	+	+	+
d-Maltose	+	+	+	+	+	+	d	+	+	+
d-Lactose	d	+	d	+	d	−	d	+	+	−
d-Melibiose	**+**	−	−	−	−	−	−	−	−	−
d-Sucrose	+	+	+	+	+	+	+	+	+	−
d-Trehalose	+	+	+	+	+	+	d	+	+	+
d-Raffinose	**+**	−	−	−	−	−	−	−	−	−
Gentobiose	+	+	+	+	+	+	d	+	+	+
l-Fucose	+	+	−	−	−	−	−	−	−	−
d-Arabitol	+	d	−	−	−	−	−	+	+	+
Potassium gluconate	+	+	+	d	+	−	−	+	+	+
Potassium 2-ketogluconate	d	−	−	−	−	−	−	+	−	−
Potassium 5-ketogluconate	+	+	+	d	d	−	−	+	+	+
Lipase activity	−	+	+	−	−	−	−	−	+	+
Motility at 28 °C	+	+	+	+	d	−	−	−	+	−

## Genome features

All isolates were grown in TSA (Fort Richard, Auckland, NZ) at 28 °C for 24 h±2 h, prior to genomic DNA extraction using either the Qiagen DNeasy blood and tissue kit, or the QiaSymphony DSP DNA Mini kit (Qiagen, Hilden, Germany) or the Chemagic^™^ 360 extraction platform (PerkinElmer, Waltham, MA, USA). The DNA quality and concentration were determined using PicoGreen (Quant-iT; Thermo Fisher Scientific). Sequencing libraries containing 1 ng of DNA were prepared using the Nextera XT chemistry (Illumina, San Diego, CA, USA) for 150 bp paired-end sequencing on an Illumina MiSeq or NextSeq sequencer, according to the manufacturer’s recommendations (Illumina). Sequence quality checks, *de novo* assembly and species identification were performed using an in-house pipeline comprising Fastp v.0.20.1 [20] (no trimming of reads was performed), Centrifuge v.1.0.4 [21], Skesa v.2.3.0 [22] and Quast v.5.0.2 [23]. Assembly statistics including depth, number of contigs, N50 and GC% are provided in Table S1, available in the online Supplementary Material.

Oxford nanopore sequencing was performed on NZRM 4767^T^, as well as four other NZ ST598 isolates to obtain finished-quality genomes. Nanopore sequencing was performed using the native barcoding kit (SQK-NDB114, Oxford, UK), run on a GridION flow cell (R10.4.1). Reads were base called using Dorado v.0.7.0 (https://github.com/nanoporetech/dorado) using the super-accurate base calling model (dna_r10.4.1_e8.2_400bps_sup). Quality trimming was performed using Filtlong v. 0.2.1 (https://github.com/rrwick/Filtlong), removing reads less than 2,000 bp and retaining 90% of the best reads. Nanopore reads were assembled using Trycycler v.0.5.4 [24], followed by long-read polishing with Medaka v.1.8.0 (https://github.com/nanoporetech/medaka) and further polishing with Illumina data using Polypolish v.0.6.0 [25].

After assembly, a circular finished-quality chromosome of 4,723,104 bp was generated for NZRM 4767^T^ (Accession NZ_CP170755). CheckM (version 1.2.3) was used to evaluate the quality of the whole-genome sequencing [26] which revealed 99.35% completeness and 1.31% contamination, thus meeting the minimum standards for prokaryotic taxonomy [27]. Two putative plasmids of 68,836 and 5,481 bp were assembled. Some variation in chromosome and plasmid length was observed among the other strains sequenced with nanopore, with chromosome sizes ranging from 4,606,130 to 4,743,899 bp and plasmids ranging from 4,359 to 261,589 bp. Annotation using Bakta v.1.5.0 [28] identified 4,404 genes of which 4,236 were coding.

All assemblies had 8 copies of 5S rRNA genes, 7 copies of 23 rRNA genes and 80 tRNA genes. Seven 16S rRNA copies were present, and these could be split into two sequence types with four copies in one type and three in the other type. Pairwise identity within each type was >99.9% and 98.5–98.8% between the two types. The most common 16S rRNA gene allele from NZRM 4767^T^ (NCBI accession: PQ431197) was extracted from the genome for analysis. The full-length 16S rRNA gene was queried in the EzBioCloud 16S database as previously described [27,29]. It is recommended that only species with 98.7% or higher 16S similarity are selected for further calculation of the overall genome-related index [27]. As outlined in Table 3, the 16S rRNA gene for NZRM 4767^T^ showed the closest similarity (98.36%) to *Y. hibernica* and *Y. artesiana*, followed by *Y. enterocolitica* subsp. *palearctica* (98.29%) and *Y. enterocolitica* subsp. *enterocolitica* (97.88%)

**Table 3.** Pairwise similarities of the 16S rRNA gene for *Y. fenwicki* NZRM 4767^T^ (NCBI accession: PQ431197) to other type strains of *Yersinia*. Nucleotide mismatch is based on differences between the *Y. fenwicki* 16S rRNA gene to the reference sequences

Name	Type strain	Accession	Pairwise similarity (%)	Mismatch/total nt
*Y. hibernica*	CFS1934	MK129259	98.36	24/1,465
*Y. artesiana*	IP42281	LR745664	98.36	24/1,465
*Y. enterocolitica* subsp. *palearctica*	Y11	FR729477	98.29	25/1,465
*Y. enterocolitica* subsp. *enterocolitica*	ATCC 9610	JPDV01000006	97.88	31/1,465
*Yersinia massiliensis*	CCUG 53443	CAKR01000050	97.75	33/1,465
*Yersinia alsatica*	IP38850	LR745670	97.68	34/1,465
*Yersinia canariae*	SRR7544370	MN434982	97.41	38/1,465
*Yersinia vastinensis*	IP38594	LR745669	97.27	40/1,465
*Y. proxima*	IP37424	LR745667	97.27	40/1,465
*Y. intermedia*	ATCC 29909	AF366380	97.26	40/1,461
*Yersinia rohdei*	ATCC 43380	ACCD01000072	97.20	41/1,465
*Yersinia nurmii*	CIP 110231	CPYD01000031	97.20	41/1,465
*Yersinia frederiksenii*	ATCC 33641	JPPS01000006	97.13	42/1,465
*Yersinia thracica*	IP34646	LR745665	97.13	42/1,465
*Yersinia mollaretii*	ATCC 43969	AF366382	97.12	42/1,461
*Yersinia pekkanenii*	CIP 110230	CWJL01000114	97.00	44/1,465

The ANI values were calculated using fastANI version 1.33 [30]. As outlined in Table 4, NZRM 4767^T^ was most closely related to *Y. enterocolitica* subsp. *enterocolitica* (94.1% ANI), *Y. enterocolitica* subsp. *palearctica* (93.9%), *Y. artesiana* (93.0% ANI) and *Y. proxima* (92.7% ANI). These ANI values were representative of all ST598 genomes analysed in the current study (Table S2). The ANI values for NZRM 4767^T^ were below the threshold of ≤95% ANI and showed a strong and clear demarcation when compared to the type strains of other *Yersinia* species. This observation is concordant with the species status of this strain according to the proposed delineation cut-off of 95–96% [27,31]. In addition, the digital DNA–DNA hybridization (dDDH) values for NZRM 4767^T^ were calculated using the recommended d_4_ formula within Type (Strain) Genome Server (TYGS) hosted by the DSMZ [32]. As outlined in Table 4, the output resulted in the comparison of 12 *Yersinia* species with dDDH d_4_ formula values ranging from 56.1% for *Y. enterocolitica* subsp. *enterocolitica* ATCC 9610^T^ to as low as 21.9% for *Yersinia ruckeri* ATCC 29473^T^, which were all below the threshold of 70% for the same species identification, further supporting NZRM 4767^T^ as a novel species. As per TYGS recommendations, dDDH formula d_4_ was used for comparisons because it is independent of genome length and thus robust against the use of incomplete draft genomes [33,35], such as *Y. enterocolitica* subsp. *enterocolitica* ATCC 9610^T^. The use of the d_4_ formula and 70% cut-off to delineate other novel *Yersinia* species has been previously used in the description of new *Yersinia* species [19,36, 37].

**Table 4.** ANI and dDDH values for *Y. fenwicki* NZRM 4767^T^ (Accession NZ_CP170755) queried against type strains of other *Yersinia* species. The dDDH values (d_0_, d_4_ and d_6_) and Confidence Intervals (model C.I.) calculated using the TYGS

*Yersinia* species, strain	ANI (%)	dDDH values		
		d_0_ % [C.I.]	d_4_ % [C.I.]	d_6_ % [C.I.]
*Y. enterocolitica* subsp. *enterocolitica* ATCC 9610^T^	94.13	74.5 [70.6–78.2]	56.1 [53.4–58.8]	73.0 [69.5–76.2]
*Y. enterocolitica* subsp. *palearctica* Y11^T^	93.92	71.0 [67.1–74.7]	55.0 [52.4–57.8]	69.7 [66.3–73.0]
*Y. artesiana* IP42281^T^	92.97	71.1 [67.2–74.8]	49.4 [46.8–52.0]	68.1 [64.7–71.3]
*Y. proxima* IP37424^T^	92.66	72.2 [68.3–74.6]	48.6 [46.0–51.2]	68.7 [65.3–72.0]
*Y. thracica* IP34646^T^	87.57	60.0 [56.3–63.6]	33.0 [30.6–35.6]	52.8 [49.7–55.9]
*Yersinia rochesterensis* ATCC BAA-2637^T^	87.55	61.3 [57.6–64.9]	33.0 [30.5–35.5]	53.7 [50.6–56.8]
*Yersinia kristensenii* subsp. *kristensenii* ATCC 33638^T^	87.52	60.1 [56.4–63.7]	32.7 [30.2–35.2]	52.7 [49.6–55.8]
*Y. canariae* NCTC 14382^T^	87.35	65.0 [61.2–68.6]	32.5 [30.1–35.0]	56.1 [53.0–59.3]
*Y. hibernica* CFS1934^T^	86.14	59.1 [55.5–62.6]	30.2 [27.8–32.7]	50.7 [47.7–53.8]
*Y. alsatica* IP38850^T^	85.31	53.4 [49.9–56.8]	28.1 [25.7–30.6]	45.7 [42.6–48.6]
*Y. frederiksenii* ATCC 33641^T^	85.27	53.5 [50.0–56.6]	27.7 [25.4–30.2]	45.5 [42.2–48.6]
*Y. rohdei* ATCC 43380^T^	84.41	46.7 [43.3–50.1]	27.2 [24.9–29.7]	40.6 [37.7–43.7]
*Y. ruckeri* ATCC 29473^T^	81.16	22.6 [19.3–26.2]	21.8 [19.6–24.3]	21.5 [18.7–24.5]

**Fig. 1.**
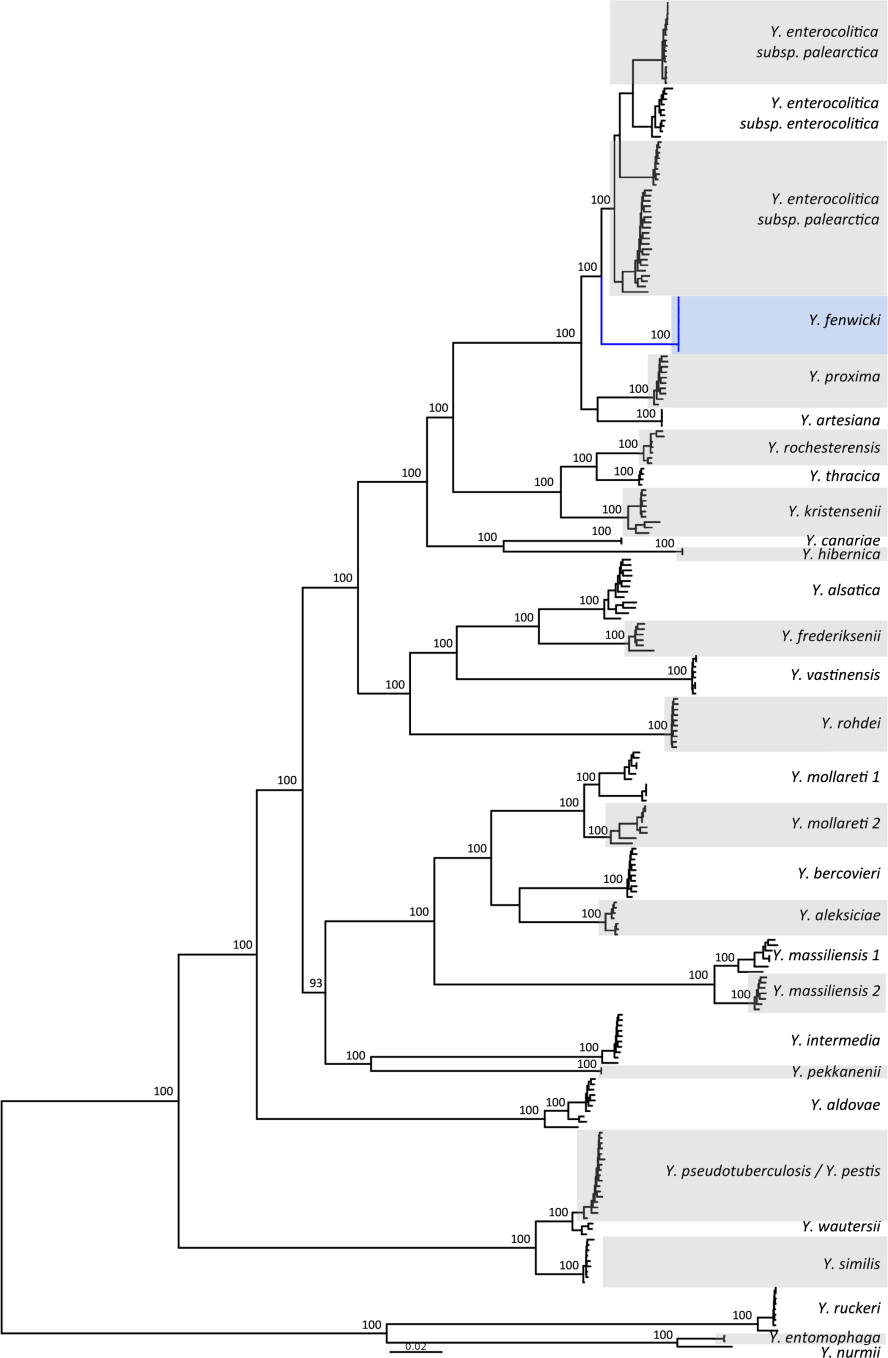
Maximum likelihood tree of *Yersinia* based on an alignment of concatenated allele sequences from 500 core genes. Bootstrap values for species-level clades are shown. The scale bar shows nucleotide substitutions per site.

Whole-genome sequencing data used to infer a multi locus ST identified these isolates as ST598 using the McNally scheme [12]. The Illumina-sequenced genome for NZRM 4767^T^, as well as all associated ST598 genomes analysed in this study (Table 1), was compared with 241 published *Yersinia* sequences [16,38]. Assemblies for the published isolates were downloaded from either the Bacterial Isolate Genome Sequence Database (BIGSdb) *Yersinia* database hosted by the Pasteur Institute [38,39] or EnteroBase [13]. Phylogenetic analysis was performed using the 500 core genes of the *Yersinia* cgMLST scheme previously described by Savin *et al*. [38]. The Genome Comparator plugin (v.2.7.7) from BIGSdb-Pasteur was used to obtain the cgMLST allele sequences for each isolate. Allele sequences were concatenated and aligned using MAFFT, and a maximum likelihood phylogenetic tree was constructed using IQTree, using the GTR+F+I+G4 model and 2,000 bootstrap replicates. The genomes for NZRM 4767^T^ and all associated ST598 genomes fell into a new clade that was strongly demarcated from other recognised *Yersinia* species (Fig. 1). All ST598 genomes analysed in this study had a very close genetic relationship with a mean ANI of 99.94% (Table S2) and 2–12 out of 500 core-allele differences between them (data not shown).

NZRM 4767^T^ and the associated ST598 genomes from NZ were analysed for virulence genes using Abricate v.1.0.1 (https://github.com/tseemann/abricate) with the virulence factor database (VFDB) [40]. All genomes lacked the chromosomally encoded *ail* virulence gene commonly associated with pathogenic BTs of *Y. enterocolitica* but did possess the *invA* gene [41] which is seen in all NZ YE including BT 1A (unpublished data). All genomes also lacked the y*stB* or *ystA* genes, with only one of these genes commonly observed in different YE BTs [41,42]. The *Y. fenwicki* described here were isolated from human clinical cases of acute gastroenteritis. However, presence does not equal causation, and it is beyond the scope of this study to establish the pathogenicity of this species.

For NZRM 4767^T^, two plasmid replicon signatures (ColRNAI_rep_cluster_1987 and rep_cluster_904) were identified with MOB-recon from MOB-suite version 3.1.0 [43]. blast analysis of the plasmids revealed no close similarities to the pYV virulence plasmid of *Yersinia* [41,44]. All ST598 genomes possessed the antimicrobial resistance genes *blaA* and *vatF* which have been previously reported in NZ *Y. enterocolitica* strains [45].

## Description of *Yersinia fenwicki* sp. nov.

*Yersinia fenwicki* (fen.wick’i, N.L. gen. n. fenwicki, named to honour and acknowledge the significant pioneering contribution made by Emeritus Professor Stanley Fenwick to yersiniosis knowledge and understanding in NZ).

Cells are short Gram-negative rods that grow well aerobically on TSA at 25–37 °C producing 1–2 mm diameter colonies after 24 h incubation. Colonies on CIN are pin-sized and circular and have a deep red centre surrounded by a translucent border. The strains tested are motile, reduce nitrate and are oxidase- and lipase-negative and catalase-positive. In API 20E tests, all strains are positive for β-galactosidase and urease utilisation, as well as indole production, and negative for lysine and ornithine decarboxylation, arginine dihydrolase, H_2_S production, tryptophan deaminase and gelatinase. In all API 50 CH tests, fermentation of l-arabinose, d-ribose, d-xylose, d-galactose, d-glucose, d-fructose, d-mannose, l-sorbose, inositol, d-mannitol, d-sorbitol, *N*-acetylglucosamine, arbutin, aesculin, salicin, d-cellobiose, d-maltose, d-melibiose, d-sucrose, d-trehalose, d-raffinose, gentobiose, l-fucose, d-arabitol, gluconate and potassium 5-ketogluconate was positive, while negative for erythritol, d-arabinose, l-xylose, d-adonitol, methyl β-d-xylopyranoside, l-rhamnose, dulcitol, methyl α-d-mannopyranoside, methyl α-d-glucopyranoside, amygdalin, inulin, d-melezitose, glucogen, xylitol, d-turanose, d-lyxose, d-tagatose, d-fucose, l-arabitol and starch.

The type strain, NZRM 4767^T^=DSM 120367^T^, and all associated ST598 isolates analysed in this study were isolated from human faecal samples from either NZ or Australia (Table 1).

The DNA G+C content of NZRM 4767^T^ is 47.01 mol%, and the chromosomal length is 4,723,104 bp. Two putative plasmids of 68,836 and 5,481 bp were also identified.

The complete genome of NZRM 4767^T^ and raw sequence reads for all isolates analysed in this study are available on NCBI SRA under BioProject numbers PRJNA1142067 and PRJEB83896 (accession numbers available in Table 1). This BioProject also includes the sequences for the two putative plasmids (accession numbers CP170756 and CP170757) identified for NZRM 4767^T^.

## Supplementary material

10.1099/ijsem.0.007034 Uncited Supplementary Material 1.

## Abbreviations

ANI: average nucleotide identity
BIGSdb: Bacterial Isolate Genome Sequence Database
BTs: biotypes
CIN: Cefsulodin-Irgasan-Novobiocin
dDDH: digital DNA–DNA hybridization
DSMZ: Deutsche Sammlung von Mikroorganismen und Zellkulturen
ERL: Enteric Reference Laboratory
NCBI: National Center for Biotechnology Information
NZ: Aotearoa | New Zealand
NZRM: New Zealand Reference Culture Medical Section
SRA: Short Read Archive
ST: sequence type
TSA: Tryptone Soya Agar
TYGS: Type (Strain) Genome Server
VFDB: virulence factor database
